# Prunin: An Emerging Anticancer Flavonoid

**DOI:** 10.3390/ijms26062678

**Published:** 2025-03-16

**Authors:** Juie Nahushkumar Rana, Sohail Mumtaz

**Affiliations:** 1 Fels Cancer Institute for Personalized Medicine, Lewis Katz School of Medicine, Temple University, Philadelphia, PA 19140, USA; ranajuie06@gmail.com; 2 Department of Chemical and Biological Engineering, Gachon University, 1342 Seongnamdaero, Sujeong-gu, Seongnam-si 13120, Republic of Korea

**Keywords:** prunin, anticancer, flavonoid, natural compounds, nanoformulation, combination therapy

## Abstract

Despite the substantial advances in cancer therapies, developing safe and effective treatment methodologies is critical. Natural (plant-derived compounds), such as flavonoids, might be crucial in developing a safe treatment methodology without toxicity toward healthy tissues. Prunin is a flavonoid with the potential to be used in biomedical applications. Prunin has yet to undergo thorough scientific research, and its precise molecular mechanisms of action remain largely unexplored. This review summarizes the therapeutic potential of prunin for the first time, focusing on its underlying mechanisms as an anticancer compound. Prunin has gained significant attention due to its antioxidant, anti-inflammatory, and anticancer effects. This review aims to unlock how prunin functions at the molecular level to exert its anticancer effects, primarily modulating key cellular pathways. Furthermore, we have discussed the prunin’s potential as an adjunctive therapy with conventional treatments, highlighting its ability to strengthen treatment responses while decreasing drug resistance. Moreover, the discussion probes into innovative delivery methods, particularly nanoformulations, that might address prunin’s bioavailability, solubility, and stability limitations and optimize its therapeutic application. By providing a comprehensive analysis of prunin’s properties, this review aims to stimulate further exploration of using prunin as an anticancer agent, thereby progressing the development of targeted, selective, safe, and effective therapeutic methods.

## 1. Introduction

### 1.1. Flavonoids and Their Significance in Biomedical Applications

Flavonoids are polyphenolic compounds synthesized in plants as bioactive with significant biological activities [1,2]. The flavonoids are responsible for various color, flavor, and pharmacological activities [1,3]. These compounds comprise 15 carbon atoms arranged into two aromatic rings coupled by a three-carbon linkage. Based on disparities in the structure of the C-ring, they are classified into distinct subgroups, including flavones, flavanones, isoflavones, flavanols, flavan-3-ols, and anthocyanidins [4,5,6]. Plants contain flavonoids that function as secondary metabolites, fulfilling essential roles like protection against UV radiation, pathogens, and herbivores [7,8]. In the inflammatory process, flavonoids can operate in several ways, including acting as antioxidants, scavenging reactive oxygen species (ROS), or decreasing free radical accumulation [9]. It also acts as an inhibitor of the activity of some regulatory enzymes like protein kinases and phosphodiesterase [10]. They modulate the activity of the immune cells (e.g., inhibition of cell activation, maturation, signaling transduction, and secretory processes). Some flavonols (e.g., quercetin, rutin, and morin), flavanones (e.g., hesperetin and hesperidin), flavonols (e.g., catechin), isoflavones (e.g., genisten), and anthocyanins (e.g., cyanidin) have exhibited anti-inflammatory functions during in vitro and in vivo experiments, as well as clinical studies [11,12]. The anti-inflammatory properties of flavonoids and their relevant compounds also significantly affect cancer development. These compounds show their activity by deactivating carcinogens, inducing apoptosis, triggering cell cycle arrest, and inhibiting angiogenesis through various pathway activation [13]. Identifying natural flavonoids as an effective and safer source of antioxidants opens new perspectives to explore more of these compounds, focusing on new structures using the latest methodologies and technologies and exploiting other new natural sources.

Prunin, a flavonoid glycoside, is a bioactive compound present in many plants and fruits [14]. It has attracted considerable interest due to its potential anticancer effects, including promoting apoptosis, inhibiting cell growth, and preventing tumor development by modulating essential signaling pathways [15]. Like other extensively researched flavonoids, prunin is anticipated to show a positive safety profile owing to its natural origins and structural resemblance to non-toxic flavonoids [16,17]. This selective toxicity probably stems from prunin’s capability to influence certain molecular pathways in cancer cells while sparing normal cells from adverse effects [18,19]. These attributes position prunin as a promising subject for future cancer prevention and treatment research [20,21,22,23].

### 1.2. Introduction to Prunin: Sources and Structure

Prunin is a flavonoid glycoside primarily found in plants, especially those from the Prunus genus, and is integral to plant growth, development, and defense systems [24,25]. Prunin is mainly found in citrus fruits and in plants belonging to the Prunus genus, including cherries and plums [15,22,26]. These bioactive compounds are produced via the phenylpropanoid pathway, a complex metabolic method including enzymes like chalcone synthase and flavanone 3-hydroxylase [27], which convert phenylalanine into flavonoid precursors [28,29,30,31]. Cherries and plums, for example, are considered some of the richest natural sources of prunin [32,33]. The most common sources of prunin include cherries (*Prunus avium* and *Prunus cerasus*) [34], plums (*Prunus domestica*) [35], peaches (*Prunus persica*), apricots (*Prunus armeniaca*), and almonds (*Prunus dulcis*) [36]. These fruits, especially their seeds and skins, contain significant concentrations of prunin, contributing to their antioxidant, anti-inflammatory, and potential anticancer properties. Besides cherries, other fruits in the Prunus family, such as blackberries and nectarines, may contain prunin, albeit in varying amounts [36]. Fruits are not the only sources of prunin; some medicinal plants within the Prunus genus or related families also contain prunin, which is used in traditional medicine for treating various diseases due to its therapeutic properties [26,36,37].

Prunin represents a broad spectrum of biological activities, such as antioxidant, anti-inflammatory, neuroprotective effects, and anti-diabetic [38]. Recent progressions in extraction and analytical techniques have enabled the isolation and characterization of prunin and other flavonoids, allowing scientists to explore their biomedical effects [39,40,41]. Monoglycoside flavonoid prunin (4,5,7-trihydroxyflavanone-β-D-glucoside) is a hydrolysis product of naringin, and the water solubility of prunin is 7.6-fold that of naringin at 25 °C, which has various biological activities [42,43]. Various methods for prunin production from different sources have been studied, including plant extraction, enzymatic techniques, and microbial and chemical synthesis [44,45].

**Figure 1:**
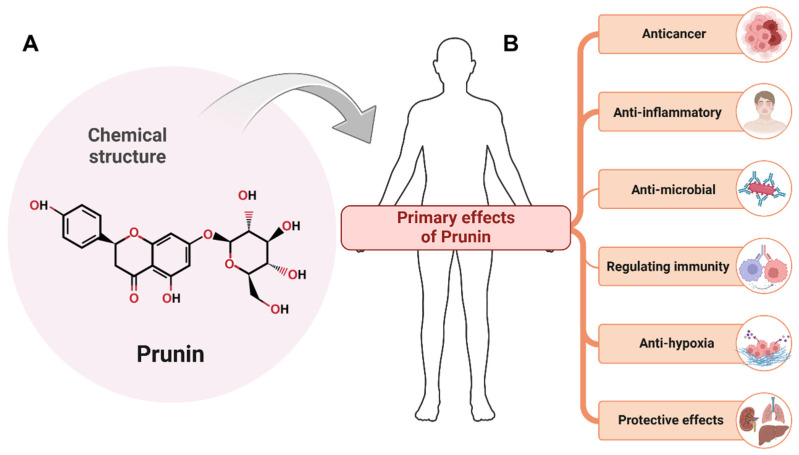
The chemical structure of Prunin and its primary biological effects. (**A**) Chemical structure of prunin. (**B**) Primary biological effects of prunin. Prunin demonstrates various pharmacological properties, including anticancer activity, anti-inflammatory effects, antimicrobial effects, immune regulation, anti-osteoporosis, anti-hypoxia, and protective effects. These benefits highlight the potential of Prunin as a versatile therapeutic compound. The figure was prepared using Biorender (BioRender.com accessed on 13 March 2025).

Prunin, a flavanone glycoside, features a chemical structure characterized by a flavanone backbone accompanied by a benzopyran skeleton [20,46]. This skeleton is typically segmented into three rings: the A-ring (the leftmost phenolic ring that contains the hydroxyl (OH) group), the B-ring (the rightmost phenolic ring), and the C-ring (the central pyran ring comprising an oxygen atom), as illustrated in Figure 1A. The structure also features several OH groups on the A and B rings, which enhances its antioxidant properties [20]. Additionally, a sugar moiety, like rhamnose or a similar glycoside, is connected to the C-ring, aiding its interaction with cellular targets [47,48,49].

Prunin’s selective toxicity toward cancer cells, as demonstrated by in vitro studies, is linked to its capacity to influence particular molecular pathways that are more active in tumor cells [48,50]. The hydroxyl groups on the B ring engage in hydrogen bonding and π-π stacking interactions with proteins and enzymes that are overexpressed in malignant cells, including tyrosine kinases and topoisomerases, eventually disrupting their functionalities [51,52,53]. In contrast, the glycosylated version of prunin appears to reduce its toxicity towards normal cells, likely because of reduced membrane permeability and a lower affinity for non-cancerous cellular targets. This characteristic is similar to that seen in other glycosylated flavonoids, including naringin and hesperidin [54,55]. This selective action is also reinforced by prunin’s structural resemblance to other non-toxic flavonoids, which are naturally occurring compounds that the human body metabolizes efficiently with no or negligible adverse effects [56,57,58].

Prunin’s safety profile is supported by its natural source, usually obtained from citrus fruits [59,60,61], and its structural similarity to well-researched flavonoids like quercetin and kaempferol [62,63,64], which demonstrates low toxicity in normal cells when used at therapeutic levels. The existence of sugar moiety may help lower the risk of oxidative damage and off-target effects, as glycosylation frequently stabilizes flavonoid structures and adjusts their reactivity [65,66]. These structures and mechanistic insights, drawn from a review of the current literature, highlight prunins’ potential as a safe and selective anticancer agent, justifying further research and concentration optimizations at various cancer targets.

Prunin is a flavonoid glycoside that assists as the glucoside derivative of prunetin [67]. The structure of prunin features a flavone backbone with a glucose molecule linked to the hydroxyl group at the C-7 position, as shown in Figure 1. This glycosylation increases its solubility and bioavailability [68], which is essential for its medical use. The biosynthesis of prunin encompasses the enzymatic conjugation of glucose to prunetin [69]. The plant’s enzymatic machinery mainly regulates the synthesis of prunin compounds [70]. The formation of prunin in plants is considered an adaptive mechanism that increases the plant’s ability to decrease or prevent oxidative damage [71].

Bioavailability refers to how rapidly a compound enters systemic circulation and reaches its target site in the body [72]. For prunin, this bioavailability is affected by its chemical structure, particularly its glycosylated form. As a flavonoid glycoside, prunin’s glucose component increases its solubility in water, which is vital for gastrointestinal absorption. Factors like enzymatic hydrolysis play a role, as gut microbiota and intestinal enzymes can convert prunin into its aglycone form (prunetin) or other metabolites [73,74]. This conversion is essential because the bioavailability of the aglycone can differ from that of prunin itself. Once absorbed, prunin and its metabolites undergo further metabolism in the liver, primarily involving phase I reactions (like oxidation and reduction) and phase II reactions (like conjugating with glucuronic acid or sulfate) [75,76]. The metabolites then circulate to various tissues, where they produce pharmacological effects [77]. Grasping prunin’s metabolic pathways and bioavailability is essential for assessing its therapeutic potential, as effective delivery to target tissues is key for its anticancer effects.

### 1.3. Importance of Prunin in Cancer Research

Cancer is known as a leading cause of death worldwide [78]. Mutations in DNA inside the nucleus of cells, caused by any exogenous or endogenous stimulant anomaly in normal cells, lead to uncontrolled division of cells, leading to tumor development and progression [79,80]. Several medications are available. For example, the most well-known chemotherapeutic agents seem to exert anti-cancer effects by assisting in cell apoptosis. In contrast, therapeutically active plant-derived compounds are regarded as marginally or not toxic while possessing significant pharmacological properties [81]. Prunin is one of the most prominent plant compounds with antioxidant and anti-cancer properties [82,83]. It has drawn considerable attention from researchers due to its structure and ability to modulate various biological pathways involved in cancer development [84]. Prunin possesses significant antioxidant activity, is vital in combating oxidative stress, and is a major carcinogen contributor [85,86,87,88]. ROS activates various apoptotic pathways for cancer treatment [88,89,90,91,92,93,94,95]. Prunin plays a crucial role in inhibiting eukaryotic translation by activating different kinases [96]. Prunin also plays a key role in scavenging ROS and inhibits many pathways like NF-κB, MAPK, STAT1, and the replication of many viruses inside the body [97,98,99,100,101]. These compounds can also inhibit the production of pro-inflammatory cytokines, which contribute to immune homeostasis [102,103]. Moreover, Prunin has substantial properties in enhancing the function of immune cells and supporting their role in both types of immunity—innate and adaptive [104]. Their therapeutic potential and significant effect on the body make them valuable candidates for immune-related interventions [104].

Plant-derived immunotherapies have emerged as a significant area of interest in modern medical research, mainly due to their potential to modulate immune responses with fewer side effects than conventional treatments [105,106]. Key findings highlight the ability of natural compounds, such as prunin, to activate both the innate and adaptive immune systems [17,107]. This compound has demonstrated capabilities in reducing inflammation, enhancing immune cell activity, and promoting antioxidant defenses, making it valuable in treating various conditions, including cancer, infections, and autoimmune disorders [108,109].

One of the most prominent challenges identified is the poor bioavailability of these plant-derived compounds [3]. Though highly active in vitro, many natural compounds are poorly absorbed in the human body, rapidly metabolized, and excreted, limiting their therapeutic potential [110]. This has led researchers to focus on innovative delivery systems like nanoparticles, liposomal encapsulation, and other advanced formulations to improve absorption, prolong circulation time, and enhance overall effectiveness [111,112]. These advancements could transform plant-derived immunotherapies from promising experimental treatments to widely used clinical options [112]. Synergy with existing treatments is another crucial finding. Plant-derived compounds can improve therapeutic outcomes when combined with conventional therapies, particularly immune checkpoint inhibitors (ICIs) [112]. These natural compounds can modulate the tumor microenvironment (TME), reduce inflammation, and enhance immune activation, making ICIs more effective and possibly lowering their side effects [113,114,115,116]. Such combinations could lead to more comprehensive and less toxic cancer treatments. Toxicological concerns are also critical. Although plant-based therapies are generally considered safer, certain compounds can cause adverse effects at high doses or with prolonged use [117,118,119]. For example, saponins may induce hemolysis, and excessive immune stimulation by some plant compounds could trigger autoimmunity or other harmful effects.

The key findings highlight the potential and the challenges associated with plant-derived immunotherapies. While the therapeutic benefits are evident, especially regarding immunomodulation and cancer treatment, additional research is necessary to refine delivery methods, guarantee safety, and comprehensively understand the underlying mechanisms. Addressing these challenges and advanced research will be essential for successfully integrating plant-derived therapies into mainstream medicine [120,121]. By exploring these mechanisms, the review aims to explain how prunin combats oxidative stress and modulates inflammation, two critical factors that contribute to cancer development. In addition to discussing prunin’s biological activities, this review summarizes preclinical studies investigating its anticancer efficacy in various cellular and animal models. Plant-derived compounds have shown immense potential in cancer treatment, offering a complementary approach to traditional therapies such as chemotherapy, radiation, and immune ICIs [122]. With their natural origins and bioactive properties, plant-based compounds can enhance the body’s immune response, modulate the tumor microenvironment, and reduce the toxic side effects of conventional treatments [123]. This has made them an area of growing interest in cancer therapy research. One of the key potential impacts of plant-derived compounds in cancer treatment is their ability to modulate the immune system. A compound such as prunin is known for its immunomodulatory effects, which can boost innate and adaptive immune responses [124]. This compound can activate immune cells, such as T-cells, natural killer (NK) cells, and macrophages, playing crucial roles in recognizing and destroying cancer cells [125,126,127,128]. Plant-based compounds can help the body target and eliminate cancer by enhancing immune surveillance and response [129,130,131].

Another significant impact of plant-derived compounds is their ability to act as adjuvants, enhancing the effectiveness of existing cancer therapies, particularly ICIs [132]. ICIs have revolutionized cancer treatment by removing the inhibitory signals that prevent the immune system from attacking tumors [132,133]. However, not all patients respond to ICIs, and many develop resistance [134,135,136,137]. Research suggests that combining ICIs with plant-derived compounds can improve the efficacy of these treatments by altering the tumor microenvironment, reducing inflammation, and enhancing immune activation [138]. This combination approach may increase the proportion of patients who benefit from ICIs and help overcome resistance. Furthermore, plant-derived compounds such as prunin can reduce the toxicity of conventional cancer therapies. Chemotherapy and radiation often cause severe side effects, including damage to healthy cells, immune suppression, and systemic toxicity [139]. With their antioxidant and anti-inflammatory properties, plant-based compounds may help mitigate these side effects [140,141,142,143]. For example, curcumin has been shown to protect normal cells from oxidative damage during chemotherapy [144], reducing its harmful effects without compromising its efficacy against cancer cells [145,146]. This ability to minimize treatment-related toxicity could improve patients’ quality of life and allow for more aggressive cancer treatment strategies without overwhelming the body.

In summary, integrating plant-derived compound prunin into cancer treatment holds considerable promise. By enhancing immune responses, improving the efficacy of existing therapies, and reducing treatment-related toxicity, these natural compounds could significantly impact how cancer is treated. Future research and clinical trials will be essential in realizing their full potential and optimizing their use in comprehensive cancer care strategies. These studies are necessary to understand prunin’s potential to target and inhibit tumor growth. To the best of our knowledge, there are currently no comprehensive reviews that specifically examine prunin’s role as an anticancer agent. This review serves as the first in-depth examination of the mechanisms through which prunin exerts its therapeutic effects, such as its capacity to modify key signaling pathways, trigger apoptosis, and hinder tumor development. By consolidating and evaluating the existing evidence, this review seeks to address a significant gap in the literature and lay the groundwork for future investigations into prunin’s potential in cancer treatment. Additionally, the review evaluates the current state of clinical research on prunin, assessing its viability as a chemo-preventive or therapeutic agent in cancer treatment. Finally, the review addresses the challenges in translating prunin’s preclinical promise into clinical application, including its pharmacokinetics, bioavailability, and the need for further clinical trials to establish its safety and efficacy. By the end of this review, we aim to provide a comprehensive framework for understanding prunin’s therapeutic potential and outline the future directions for its study in cancer therapy.

## 2. Therapeutic Potential of Prunin and Mechanism of Action in Cancer

**Figure 2:**
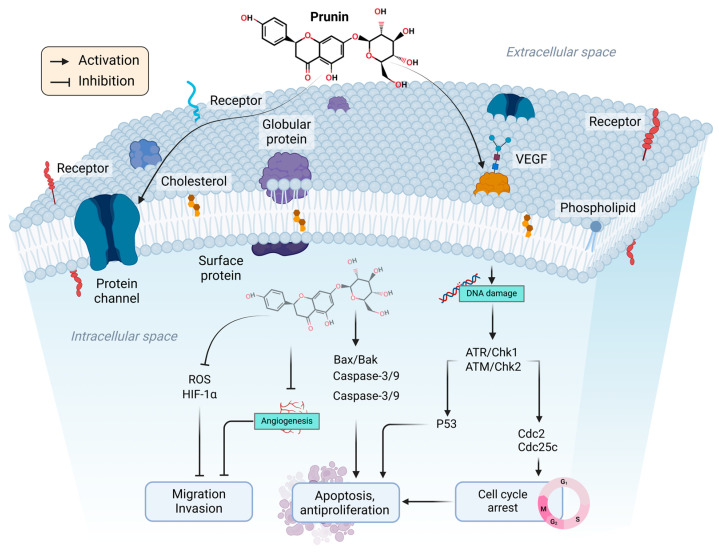
The biological significance of prunin. Prunin influences multiple pathways in tumor cells at the molecular level, ultimately inducing key therapeutic effects such as cell cycle arrest, apoptosis, antiproliferation, and anti-angiogenesis, as well as anti-metastasis and invasion. The figure was prepared using Biorender.

Prunin has garnered significant interest due to its anticancer potential, mainly because it impacts various biological pathways involved in oncogenesis [147,148]. These pathways regulate essential processes such as cell growth, cell cycle regulation, cell death, angiogenesis, and metastasis—crucial factors in cancer [149]. By regulating various pathways, prunin suppresses cancer growth and induces apoptosis. Its multifaceted action underscores its broad-spectrum effects, offering a deeper understanding of its capability to combat cancer at a molecular level [150]. Figure 2 demonstrates that prunin induces apoptosis by influencing multiple molecular targets involved in cellular and receptor-mediated cell death pathways [151]. Thus, targeting these apoptotic pathways offers a hopeful strategy for fighting cancer.

### 2.1. Effect of Prunin on Cell Cycle

**Figure 3:**
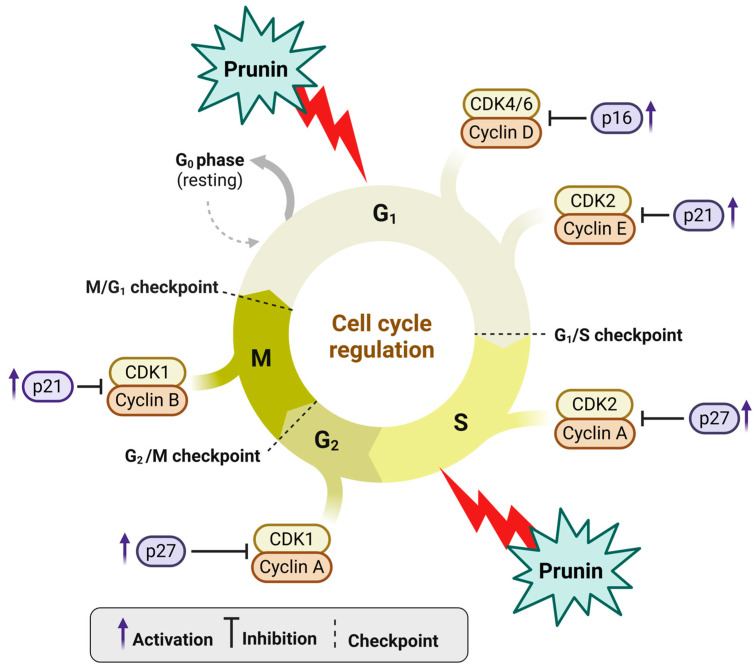
Prunin influences cell cycle regulation by interacting with various CDKs and cyclins. Prunin is helpful for modifying CDKs and cyclins during different cycle phases. It can inhibit CDK4, CDK6, and cyclin D action by increasing the expressions of the p21 marker in the G_1_ phase. At the G_1_/S checkpoint, prunin suppresses CDK2-Cyclin E by increasing p21 expressions. Similarly, CDK2-Cyclin A activity is restricted by the p27 marker [166,167,168]. This highlights the potential of flavonoids such as prunin to influence or induce cell cycle arrest, which contributes to cancer treatment. The figure was prepared using Biorender.

One of the primary ways the prunin exerts its anticancer effects is by influencing the cell cycle [152,153], as shown in Figure 3. The cell cycle is a regulated process that includes several checkpoints (G1/S, G2/M, and M/G1 checkpoints) and regulatory proteins that ensure proper cell division [154,155]. Cell cycle dysregulation can lead to uncontrolled cell growth and eventually induce cancers [154,155]. On the other hand, the arrest of the cell cycle can be used to inhibit cancers [156]. Prunin impacts cell cycle regulation through interactions with various cyclin-dependent kinases (CDKs) and cyclins, as illustrated in Figure 3. Prunin has been demonstrated to influence crucial cell cycle regulators, including cyclins and CDKs [157,158]. Cyclins are essential proteins that regulate CDKs, which phosphorylate target proteins to advance the cell cycle [158]. Excessive cyclin and CDK expression lead to uncontrollable cell division, frequently seen in cancers [159]. Prunin alters the expression levels of several cyclins, especially cyclin D1 and cyclin E, along with their related CDKs, like CDK4 and CDK2 (see Figure 3). By inhibiting these cyclins and CDKs, prunin effectively triggers cell cycle arrests at vital checkpoints, suppressing cancer cells or initiating apoptosis [160]. The G1/S and G2/M phases serve as essential checkpoints in the cell cycle, where the cell primarily determines whether to continue with DNA replication or proceed to mitosis. Prunin has the capability to induce cell cycle arrest at these two points [161,162,163]. During the G1/S phase, prunin can inhibit cyclin D-CDK4 complex activity, activating the retinoblastoma protein (pRb) [164,165]. This activation stops the cell from moving into the S phase. In the G2/M phase, prunin can impact cyclin B-CDK1 complex activity, blocking the cells from beginning mitosis [152]. This dual-phase cell cycle arrest at G1/S and G2/M stages caused by prunin effectively decreases or inhibits tumor growth.

### 2.2. Intrinsic and Extrinsic Pathways

**Figure 4:**
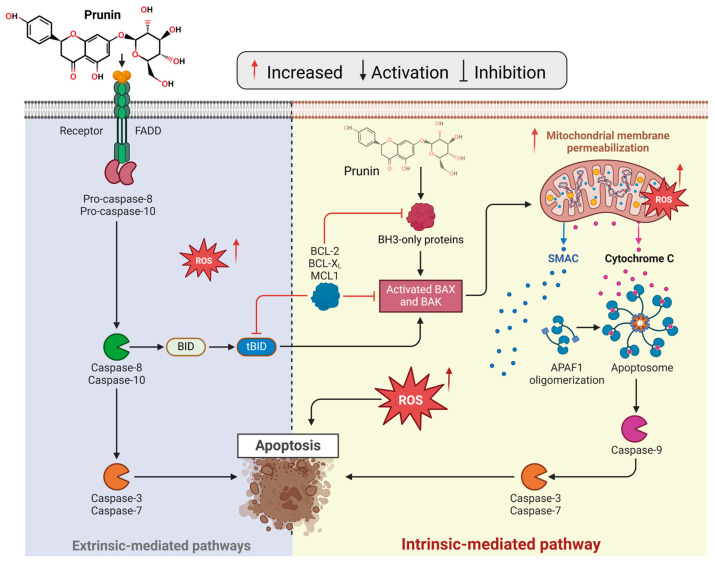
The prunin compound induces apoptosis by triggering intrinsic and extrinsic-mediated pathways. In the extrinsic pathway, the prunin compound activates the FAS receptor, triggering FADD and cleaving pro-caspase-8/10. The activated caspase-8 processes BID into tBID, effectively linking the extrinsic pathway to the intrinsic pathway by enhancing the permeabilization of the outer mitochondrial membrane. In the intrinsic pathway, prunin can cause mitochondrial dysfunction by influencing endogenous ROS levels and altering the balance between pro-apoptotic and anti-apoptotic proteins [172,174,175]. The figure was prepared using Biorender.

Cellular stresses like DNA damage or oxidative stress can introduce the intrinsic apoptosis pathway [169], releasing pro-apoptotic proteins from mitochondria. Prunin activates the intrinsic pathway by enhancing mitochondrial membrane permeability, eventually triggering the release of cytochrome c and introducing caspase cascades [170], as illustrated in Figure 4. Prunin can affect intrinsic and extrinsic apoptotic pathways to manage cancer cell death effectively [170]. It upregulates the expression of BAX and BAK genes while downregulating BCL-2, BCL-XL, and MCL1 in the intrinsic pathway [171,172]. Prunin can promote autophagy by blocking the AKT/mTOR/p70S6K pathway, inhibit the development of ovarian cancer cells by activating PARP-1 and caspase-9, and induce apoptosis to treat malignancies [173].

Like other flavonoids, prunin can prevent cell necrosis mainly by lowering mitochondrial ROS production, preserving ATP levels, preventing oxidative harm, and releasing mitochondrial DNA [176]. In contrast, the extrinsic pathway is initiated by activating death receptors on the cell’s surface [177]. Prunin boosts the expression of death receptors like Fas and TRAIL (TNF-related apoptosis-inducing ligand) [176,178,179], thus triggering the extrinsic apoptotic pathway (Figure 4). Caspases are a group of cysteine proteases essential for the process of apoptosis [180]. Prunin stimulates the activation of several caspases, such as caspase-3, caspase-8, and caspase-9, resulting in the cleavage of crucial cellular pathways and the elevation of cell apoptosis [181]. This modulation of the cascade by prunin ultimately causes cell death while inhibiting tumor progression. Conversely, a key mechanism of mitochondria-independent, or extrinsic, apoptosis induction by prunin involves its effects on death receptors [181]. These receptors are essential for transmitting apoptotic signals from the cell membrane to the cytoplasmic signaling pathways [182]. Prunin effectively activates Nrf2, initiating antioxidant genes, and offers protection against oxidative stress in hepatocytes [182]. Prunin may protect against liver oxidative injury by activating Nrf2 and boosting cellular antioxidant responses [66].

### 2.3. The PI3K/Akt/mTOR Pathway

The PI3K/Akt/mTOR-signaling pathway is crucial and plays a key role in regulating various cellular processes [183,184]. Key proteins involved in this pathway include PI3K and AKT, which are vital to its signaling mechanisms [185,186,187]. Dysregulation of the PI3K/Akt/mTOR-signaling pathway is associated with malignancy initiation, growth, and progression [188,189,190]. Furthermore, PTEN acts as a significant negative regulator by transforming PIP3 back into PIP2, which helps inhibit the activation of the PI3K/Akt/mTOR pathway [189]. Targeting a single pathway like PI3K often leads to toxic side effects in patients. Therefore, the inhibition of the PTR pathway for oncological treatment is greatly improved by using combination techniques. For example, combining natural compounds may reduce toxicity and offer an intriguing approach for more effective targeted cancer treatments [189]. Prunin is a prospective therapeutic agent for cancer treatment since it directly targets essential components of the PI3K/Akt/mTOR pathway [191]. Autophagy is a vital cellular mechanism that breaks down damaged proteins and organelles to preserve stability [20].

In cancers, autophagy can be activated by deregulating the PI3K/Akt/mTOR pathway, permitting tumor cells to survive in environments lacking nutrients [191]. This adaptive mechanism is essential for cancer development and therapy resistance. Anticancer therapies aimed at the PI3K/Akt/mTOR pathway can promote autophagy, which inhibits mTOR, a crucial regulator [191]. mTORC1 suppresses autophagy and lysosomal activity by phosphorylating crucial proteins like ULK1, which is involved in autophagy regulation, and TFEB, which controls lysosomal gene expression. The activity of mTORC1 is influenced by energy status, hypoxia, and other conditions, affecting autophagy through the AMPK/TSC pathway [192]. Prunin can promote autophagy by inhibiting the PI3K/mTOR-signaling molecules [186,193,194,195]. Prunin blocks Akt/mTOR signaling, causing both autophagy and apoptosis [196]. Moreover, prunin can decrease LC3II levels and partially restore p62 degradation in MN9D cells, which controls autophagy.

### 2.4. Antioxidant and Anti-Inflammatory Mechanisms

Prunin’s antioxidant activity is a key feature that makes it a promising agent against diseases related to oxidative stress, such as cancer [162]. By scavenging ROS and reactive nitrogen species (RNS), prunin helps protect cellular components like DNA, proteins, and lipids from oxidative damage. This protection is essential for cancer prevention, as the accumulation of oxidative damage can lead to genetic mutations and promote the onset of cancer [34]. Prunin’s antioxidant characteristics are mainly attributed to its capability to donate electrons, neutralize free radicals, and prevent cellular damage to preserve cellular homeostasis [197,198]. A recent study by Zhang et al. showed that the prunin isolated from *Bauhinia variegate* induces a protective effect against diet-induced atherosclerosis by reducing the levels of proinflammatory mediators such as TNF-α and IL-6 [198]. In addition to its antioxidant characteristics, prunin exerts significant anti-inflammatory effects [199]. Chronic inflammation plays a key role in cancer progression, and prunin’s ability to modulate inflammatory pathways is essential in mitigating this risk. One of the significant anti-inflammatory mechanisms of prunin is its inhibition of the NF-κB-signaling pathway, which is generally triggered in several cancers. NF-κB serves as a transcription factor that governs the expression of pro-inflammatory cytokines, chemokines, and adhesion molecules, all of which play a significant role in tumor promotion [200,201,202]. By preventing NF-κB activation, prunin decreases the production of these inflammatory mediators, thereby preventing tumor progression and metastasis processes [203]. Furthermore, pruning modulates various inflammatory pathways, including MAPK (mitogen-activated protein kinase) and COX-2 (cyclooxygenase-2), further contributing to its anti-inflammatory effects [204,205,206].

Prunin decreases ROS and RNS levels by functioning as a direct antioxidant [207]. Prunin efficiently scavenges harmful radicals like superoxide, hydroxyl radicals, and hydrogen peroxide by donating electrons to neutralize them [208,209]. This phenomenon transforms highly reactive species into more stable and less harmful molecules, reducing oxidative stress. By decreasing ROS and RNS, prunin protects cells from oxidative damage. Furthermore, prunin improves the phosphorylation of key signaling molecules, indicating its protective role against oxidative stress [207]. Prunin is a therapeutic agent for improving glucose homeostasis and is involved in glucose uptake [61]. In addition to prunin’s direct scavenging capability, it also triggers the action of several key endogenous antioxidant enzymes, such as superoxide dismutase (SOD), glutathione peroxidase (GPx), and catalase (CAT) [61,210]. These endogenous antioxidant enzymes are essential in preserving cellular redox balance by converting highly reactive ROS into less reactive or bio-friendly molecules [211,212,213].

### 2.5. Activation of the P53 Pathway by Prunin

**Figure 5:**
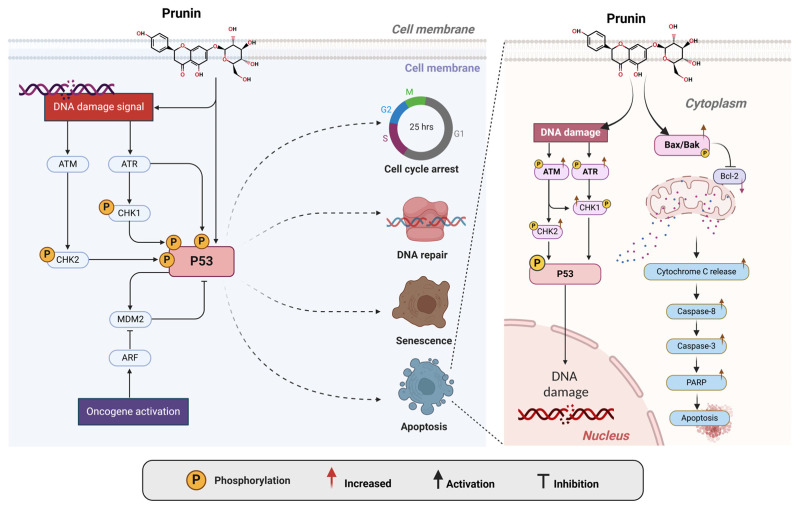
The prunin compound induces activation of P53 pathways. The prunin stimulates the P53 pathways after receiving the DNA damage signal, which further leads to multiple tasks, including cell cycle arrest, DNA repair if the damage is moderate, senescence, and apoptosis [84,226,227]. The apoptosis mechanism is highlighted when DNA damage stimulation occurs by prunin. It increases the activation of ATM, ATR, CHK1, and CHK2, which further leads to the activation of its downstream marker P53. The P53 further activates BAX and caspase cascades to induce apoptosis in cancer cells [228]. The figure was prepared using Biorender.

Primarily, many cancers are associated with inactivated P53 expression [214]. P53 is crucial in various ways inside cancer cells (Figure 5). The main biological functions of P53 in cancer cells include triggering apoptosis, regulating cellular senescence, inhibiting angiogenesis, controlling the cell cycle, modulating cellular differentiation, and maintaining DNA metabolism [215]. Within tumor cells, P53 acts as a molecular sensor that inhibits cell proliferation in response to detrimental stimulation. P53 induces apoptosis by activating the Bax gene (a key member of the Bcl-2 family) through the mitochondrial intrinsic pathway [215]. In the intrinsic pathway, Bax binds to Bcl-2, thereby activating the production of apoptotic mediators (e.g., caspase 3/9 and cytochrome C) through mitochondrial activity [215]. Thus, plant flavonoid prunin may be a valuable source to target Bcl-2 through p53, offering an efficient means for combating cancer [216]. Prunin could activate the ATM/ATR pathway by inducing DNA damage inside cells through oxidative stress or cellular homeostasis disruption, generating DNA damage signals and leading to cell death as ATR/ATM is linked with CHK1 and CHK2 cell cycle checkpoints that can be triggered through prunin, which leads these kinases to phosphorylate downstream effectors, including checkpoint kinases CHK1 and CHK2 [217,218,219,220,221]. Subsequently, it will phosphorylate the tumor-suppressor protein P53. Phosphorylation of P53 stabilizes it by avoiding its degradation through MDM2 inhibition, permitting its initiation [222]. P53 coordinates a multifaceted cellular response, promoting cell cycle arrest to allow DNA repair [223]. Purine activity inside cells induces oxidative stress, which leads to apoptosis if the damage is acute and starts cellular senescence to prevent the proliferation of damaged cells. Prunin treatment could potentially increase the expression of death receptors Fas Ligand (FasL) protein in human gastric cancer cells [223]. Additionally, it induced the activation of caspase-8 and caspase-3 and the cleavage of PARP after P53 signaling through the intrinsic pathway [223]. By initiating this pathway, purine increases genomic integrity and exerts its anticancer effects, indicating its therapeutic potential as a modulator of the DNA damage response and tumor-suppressor pathways [224,225].

### 2.6. Activation of MAPK Pathway by Prunin

Prunin, as a potential flavonoid compound, has gained significant attention due to its potential for biomedical applications for future research [22,32,46,229,230]. Prunin could be a candidate for molecular studies that modulate the mitogen-activated protein kinase (MAPK) pathway, which plays a vital role in the signaling cascade for cellular responses to external stimuli, including oxidative stress, inflammation, and oncogenic signals [231,232,233]. Prunin treatment could increase the phosphorylation and activation of MAPK and act as an anticancer agent by exhibiting pro-apoptotic activities inside cells [234]. There are three key hallmarks in the MAPK pathway, which play different roles according to situations. It comprises (subfamilies) such as ERK (extracellular signal-regulated kinase), JNK (c-Jun N-terminal kinase), and p38, which play a central role in regulating cell functions such as proliferation, apoptosis, differentiation, and stress responses [235,236,237].

In the cells, various transport systems available for prunin could enter inside cells through active or passive transport, which interacts with membrane receptors such as receptor tyrosine kinases (RTKs) or G-protein coupled receptors (GPCRs) upon cellular uptake [238]. This interaction stimulated a cascade of phosphorylation actions, stimulating Ras, a small GTPase protein. This process is conducted through stimulated Ras, which recruits and activates Raf, a serine/threonine kinase. Raf phosphorylates and activates MEK1/2, which in turn phosphorylates and activates ERK1/2 [239]. Further, ERK1/2 translocates to the nucleus, influencing numerous transcription factors and ultimately driving gene expression in cell survival, proliferation, repair processes, and apoptosis according to the signal [239]. Some experimental data show that using different plant flavonoid compounds further demonstrates that they suppress cell growth and migration and induce apoptosis via MAPK/mTOR pathway using combination treatment [240]. Prunin could inhibit the proliferation and migration of cancer cells, possibly by downregulating MAPK14 expression, which is linked to poor prognosis [241]. Therefore, plant flavonoids could be potential therapeutic drugs stemming from their ability to modulate the MAPK pathway, thereby reducing tumor growth and metastasis [242].

In addition to ERK activation, prunin could influence the JNK and p38 MAPK branches, which leads to cell death signaling. Under oxidative stress conditions, prunin mitigates ROS production, stabilizing the intracellular redox balance [243,244]. This antioxidant effect curtails the overactivation of JNK and p38 pathways in cells, avoiding excessive inflammatory responses and apoptosis. Interestingly, prunin could selectively increase JNK and p38 activity in cancer cells, endorsing pro-apoptotic signaling. Prunin accomplishes this by inducing the expression of upstream kinases, which phosphorylate JNK and p38 after treatment [245]. The selective cytotoxicity that prunin could exhibit against malignant cells underscores its therapeutic potential as an anticancer agent. It suggests that prunin could be a potential bioactive compound on the MAPK pathway [245].

Beyond prunin’s antioxidant and apoptotic roles, which play vital roles inside cells that activate several signaling pathways, it exerts potent anti-inflammatory effects by regulating MAPK-driven cytokine production [246,247]. By reducing the activation of NF-κB (a downstream effector of the MAPK pathway), prunin could inhibit the transcription of pro-inflammatory cytokines such as TNF-α, IL-6, and IL-1β [248]. This dual inhibition of MAPK and NF-κB signaling by prunin alleviates inflammation and decreases the tumor-facilitating microenvironment [169,249]. The anticancer properties of prunin are further augmented by its capability to cause cell cycle arrest and apoptosis via MAPK-mediated pathways. For instance, activating JNK by prunin improves the phosphorylation of p53, a tumor-suppressor protein that leads to the transcription of pro-apoptotic genes like BAX and PUMA [248]. Simultaneously, prunin could disrupt the phosphorylation of BCL-2, an anti-apoptotic protein, tilting the cancerous cells’ balance toward programmed cell death [250,251,252,253]. Thus, prunin, a plant bioactive compound, could be a multifaceted regulator of the MAPK pathway, showing its antioxidant, anti-inflammatory, and anticancer potential to modulate various cellular functions [254]. By targeting specific branches of the MAPK cascade context-dependently, prunin displays notable potential as a therapeutic bioactive compound for dealing with oxidative stress, chronic inflammation, and tumor prevention. Further research might be required to unravel its precise molecular interactions and optimize its clinical applications for real applications.

### 2.7. Modulation of Tumor Microenvironment (TME)

**Figure 6:**
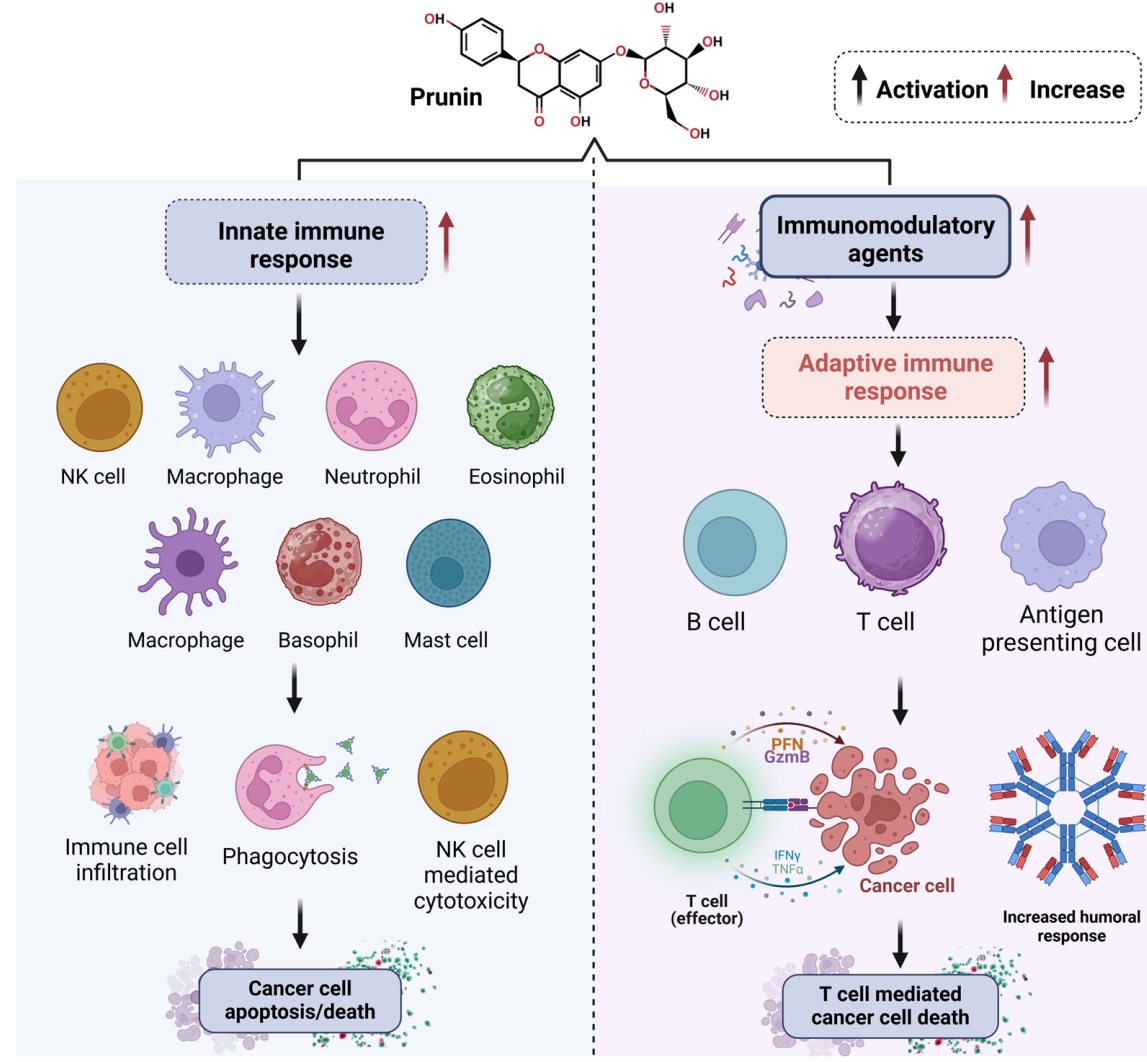
Overview of the immunomodulatory effects of prunin on innate and adaptive immune responses. Activation (black arrow), increased response (red arrow). The figure was prepared using Biorender.

Like other flavonoids, prunin can modulate immune cell influx in the TME [255,256,257]. Research shows it aids in the recruitment of tumor-suppressor cells, such as NK cells and cytotoxic T lymphocytes, while inhibiting the influx of pro-tumorigenic immune cells like tumor-associated macrophages [258,259,260,261]. This immune modulation contributes to the anti-tumor effects of prunin, supporting the body’s natural defense mechanisms against cancer development [262]. The immunomodulatory effects of isorhamnetin on the innate and adaptive immune responses are shown in Figure 6. Prunin boosts the innate immune response by stimulating various immune cells, such as NK cells, macrophages, neutrophils, eosinophils, basophils, and mast cells [262]. This stimulation promotes immune cell infiltration, enhances phagocytosis, and increases NK cell-mediated cytotoxicity, culminating in the apoptosis and death of cancer cells [263,264]. Simultaneously, prunin stimulates adaptive immunity by modulating antigen-presenting cells, B cells, and T cells [265,266]. Enhanced T cell activity promotes cancer cell death through effector mechanisms involving perforin, granzyme B, interferon-gamma, and tumor necrosis factor-alpha [267,268,269,270]. Furthermore, pruning enhances the humoral immune response, boosting antibody production for a strong immune defense [271,272].

### 2.8. Suppression of Angiogenesis and Metastasis by Prunin

Metastasis is a complex procedure that comprises the transport of tumor cells from the primary site to secondary lesions [273]. Angiogenesis is one of the key mechanisms that support cancer progression processes [273,274]. Flavonoids have shown their potential to disrupt these key cancerous phenomena (angiogenesis and metastasis) and restrict their survival and spread [275,276,277]. Vascular endothelial growth factor (VEGF) is a key pro-angiogenic factor that promotes the development of new blood vessels, which enhances the supply of oxygen and nutrients, subsequently contributing to cancer growth [278]. VEGF enhances angiogenesis by stimulating the development of new blood vessels, which are crucial for delivering oxygen and nutrients essential for tumor growth and aiding metastasis [279,280,281,282]. The inhibition of VEGF expression can restrict the formation of new blood vessels and limit the supply of oxygen and necessary nutrients for cancer cell survival, eventually leading to cell death [283]. The prunin can inhibit VEGF expression, suppressing angiogenesis and tumor vascularization [284]. Prunin inhibits cancer growth and metastases by reducing the expression of VEGF and MMP-2 while increasing endostatin levels, an angiogenesis inhibitor [285,286]. Prunin also elevates immune markers IL-2 and IFN-γ, indicating an improved immune response. Additionally, prunin reduces the expression levels of MMP-9 without cytotoxic effects, suggesting it could be an essential natural antioxidant and MMP inhibitor related to oxidative stress [287,288]. The prunin significantly reduced EMT (epithelial-mesenchymal transition) and lowered STAT3, a transcription factor that promotes tumor invasion, thereby inhibiting blood vessel formation [289,290]. Prunin inhibits several transcription factors crucial for regulating immune cells’ differentiation, proliferation, and activation while promoting T-cell generation [291]. This compound influences various immune system processes and holds potential for therapeutic applications [292].

Angiogenesis is one of the most important factors for cancer progression. Several angiogenic proteins include VEGF, essential fibroblast growth factor, IL8, and TGF-β, whereas anti-angiogenic factors include thrombospondin-1, angiostatin, and endostatin. Flavonoids such as prunin act as effective angiogenesis inhibitors and are a promising treatment for controlling malignancies [293]. Prunin exhibits its angio-inhibitory effect by decreasing VEGF and other related factors. It has also been reported to cause downregulation of the TGF-β pathway, thereby reducing metastasis and invasion [227,234]. Inflammation is a hallmark of cancer progression as the immune system plays a crucial role in combating cancers, and its chronic activation contributes to tumor growth, metastasis, and resistance to chemotherapy. Prunin’s ability to modify inflammatory mediators and signaling molecules makes it a promising candidate for cancer therapy. Additionally, prunin has demonstrated anticancer activity in various in vitro and in vivo models by inducing cell death in cancer cells [294]. It has been found to modify the expression of key proteins involved in apoptosis, such as caspases, Bcl-2 family members, and p53. Through these mechanisms, prunin may help to eliminate transformed cells while sparing normal, healthy cells [295]. Due to these promising properties, prunin is gaining recognition as a potential chemo-preventive agent. However, further clinical studies are necessary to fully establish its efficacy and safety as a therapeutic agent in cancer treatment.

## 3. Potential for Combining Prunin with Conventional Therapies

**Figure 7:**
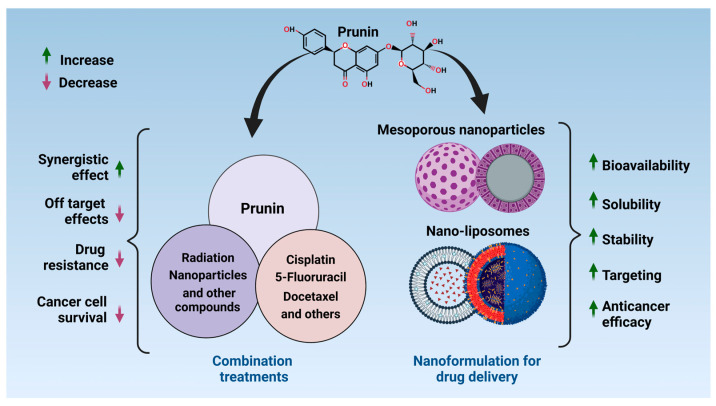
Overview of prunin for combination treatment and nanoformulation for improved drug delivery. Prunin can be utilized alongside radiation, nanoparticles, various natural compounds, and anticancer chemotherapy drugs to achieve a synergistic effect, helping to reduce drug resistance and enhance cell activity and death. For drug delivery, the nanoformulation increases the bioavailability, solubility, stability, target delivery, and overall efficiency of the prunin [310,311,312]. The figure was prepared using Biorender.

Research on flavonoid’s role in improving chemotherapy effectiveness is crucial in cancer studies [296,297]. While chemotherapy is essential for cancer treatment, it often results in side effects and drug-resistance problems [298,299,300]. The flavonoids, especially prunin, exhibit antioxidant, anti-inflammatory, and apoptotic properties that may allow them to enhance chemotherapy outcomes [301]. Prunin can make cancer cells more sensitive to chemotherapy-induced death by regulating several signaling pathways related to chemoresistance (Figure 7) [302]. For example, prunin inhibits the PI3K/Akt/mTOR pathway and promotes apoptosis in cancer cells, which could boost the cytotoxicity of chemotherapeutic drugs [303,304]. Moreover, prunin’s antioxidant characteristics might alleviate the oxidative damage that chemotherapy drugs inflict, protecting healthy cells while enabling cancer cell death. As such, combining prunin with chemotherapy might enhance treatment effectiveness, lower required dosages, and reduce the side effects typically associated with chemotherapy.

Radiotherapy is a common treatment for cancer that uses ionizing radiation to destroy or damage cancer cells [305]. Although it is effective, this therapy can also harm surrounding healthy tissues, resulting in side effects such as fatigue, skin reactions, and immunosuppression [305]. Prunin, known for its antioxidant properties [22,199], might be a beneficial supplement to radiotherapy by enhancing its effectiveness while minimizing harm to normal tissues. By neutralizing free radicals produced during radiation treatment, prunin can help protect healthy cells from oxidative stress caused by radiation, potentially reducing side effects and improving patients’ quality of life [306]. Additionally, prunin can promote apoptosis in cancer cells, increasing their susceptibility to radiation-induced cell death and enhancing treatment efficacy [307,308,309]. Research indicates that flavonoids like prunin can enhance the cytotoxicity of radiation on cancer cells by inducing DNA damage and impairing repair processes, thereby improving the overall success of radiotherapy [234]. In conclusion, when combined with standard cancer treatments like chemotherapy and radiotherapy, prunin’s synergistic effects can boost treatment effectiveness while minimizing side effects and addressing resistance mechanisms. Its diverse actions—antioxidant, apoptotic, and signaling modulation—position prunin as a promising choice for combination therapies to enhance the overall success of cancer treatment.

## 4. Nanotechnology and Drug Delivery Systems for Prunin

One significant research area in harnessing prunin’s therapeutic potential is its increase its bioavailability [313]. As a flavonoid glycoside, prunin’s absorption is restricted due to its low solubility and essential first-pass metabolism [314,315,316,317]. To address these challenges, nanotechnology offers innovative solutions by developing nanoformulations that improve prunin’s solubility, stability, and bioavailability (Figure 7) [313,318,319]. Nanoparticles like liposomes, solid lipid nanoparticles, and mesoporouss can be tailored to encapsulate prunin, protecting from degradation and easing its absorption in the gastrointestinal tract [320,321,322,323]. These nanoformulations can be formulated for controlled release, assisting in maintaining therapeutic concentrations for a long time [324,325,326,327]. Additionally, by improving prunin’s solubility and stability, these formulations can increase tissue penetration, permitting the compound to reach target sites more efficiently [328,329,330,331]. Leveraging nanotechnology to optimize prunin’s pharmacokinetics could notably boost its potential as a potent therapeutic agent for cancer treatment [332,333,334,335]. Furthermore, targeted drug delivery is a cutting-edge approach that aims to deliver therapeutic agents precisely to tumor cells, diminishing damage to normal tissues and improving treatment outcomes [336,337,338,339]. For prunin, targeted delivery systems using nanotechnology could be a breakthrough in increasing its anticancer impacts while decreasing the risk of systemic toxicity [340,341,342,343].

Beyond receptor-mediated targeting, nanoparticles can also be formulated to exploit the enhanced permeability and retention (EPR) effect, where they accumulate in tumor tissue because of the unique vasculature in tumors [344,345,346,347]. This passive targeting approach further elevates prunin concentration at the tumor site, enhancing its anticancer effectiveness [348]. By integrating active and passive targeting methods, prunin can be delivered more efficiently to cancer cells, boosting its therapeutic impact while reducing off-target effects. Creating these targeted delivery systems offers a promising pathway for enhancing the clinical use of prunin in cancer therapeutics [348]. Future research in the delivery of prunin should focus on developing multifunctional nanoparticles that improve bioavailability and targeting and allow for the simultaneous delivery of other therapeutic agents, such as chemotherapeutic drugs or RNA-based therapies [12,349]. Combining prunin with other conventional treatment agents could produce a synergistic effect, improving overall treatment methodology [350]. Advances in nanomedicine, such as using stimuli-responsive nanoparticles that release their payload in response to specific triggers (e.g., pH, temperature, or light), could also offer new ways to control prunin delivery in the TME precisely [351,352,353]. In summary, although nanotechnology shows significant potential for enhancing prunin delivery and boosting its anticancer effects, multiple challenges must be tackled. Ongoing research on designing safe, effective, and scalable nanoformulations and developing targeted delivery systems will be essential for converting prunin’s preclinical potential into successful clinical treatments.

## 5. Conclusions and Future Perspectives

### 5.1. Summary of Key Findings

Prunin has significant potential as an anticancer natural compound due to its potential to influence various biological activities, including antioxidant, anti-inflammatory, anti-angiogenesis, anti-metastasis, and pro-apoptotic activities. Prunin effectively modulates key cellular signaling pathways in cancer cell survival, proliferation, and metastasis, making it a capable candidate for cancer treatment and prevention. Remarkably, prunin inhibits the proliferation of cancer cells by regulating cyclins, while CDKs induce apoptosis through caspase activation and mitochondrial dysfunction. Prunin also inhibits metastatic progression by downregulating MMPs, VEGF, and EMT markers. Furthermore, its antioxidant and anti-inflammatory characteristics mitigate endogenous ROS and pro-inflammatory cytokines, inhibiting cancer progression and regulating the tumor microenvironment. Prunin is also a potential candidate as an adjunct to available traditional therapies, increasing their efficacy and overcoming resistance mechanisms. Advances in nanotechnology and targeted drug delivery schemes might further improve its therapeutic potential by improving bioavailability, solubility, and stability.

### 5.2. Limitations and Future Perspectives

Despite its promise of anticancer potential, numerous limitations hinder Prunin’s clinical translation. Current research lacks comprehensive in vivo studies, and toxicological data remain insufficient, restricting complete understanding. The detailed mechanisms and precise molecular targets have not been thoroughly studied, and there is a limited exploration of their efficacy in combination therapies. Few therapeutic targets were explored; no modern drug delivery systems were tested to improve bioavailability and targeted therapy. These research gaps highlight the need for more preclinical investigations.

Future studies should address these limitations through well-designed in vivo studies, detailed toxicological assessments, and underlying mechanistic studies to identify prunin’s molecular targets. Integrating artificial intelligence (AI) and machine-learning technology into prunin research could accelerate drug discovery by predicting optimized derivatives, classifying novel targets, and enhancing combination therapies. Emerging nanoformulations and personalized medicine methods will also increase prunin’s clinical applicability. Collaborative efforts between scientists, clinicians, and pharmaceutical developers are essential to translate prunin from a promising natural compound into a clinically approved therapeutic. Addressing bioavailability, scalability, and regulatory challenges is necessary for unlocking Prunin’s full anticancer potential.

## Data Availability

Data are contained within the article.

## Abbreviations

The following abbreviations are used in this manuscript:

ROS	Reactive oxygen species
ICIs	Immune checkpoint inhibitors
NK	Natural killer
VEGF	Vascular endothelial growth factor
CDK	Cyclin-dependent kinase
RNS	Reactive nitrogen species
SOD	Superoxide dismutase
CAT	Catalase
GPx	Glutathione peroxidase
MAPK	Mitogen-activated protein kinase
ERK	Extracellular signal-regulated kinase
JNK	c-Jun N-terminal kinase
RTKs	Receptor tyrosine kinases
GPCRs	G-protein coupled receptors
EPR	Enhanced permeability and retention
AI	Artificial intelligence
TME	Tumor microenvironment
